# Automatic Grading of Disc Herniation, Central Canal Stenosis and Nerve Roots Compression in Lumbar Magnetic Resonance Image Diagnosis

**DOI:** 10.3389/fendo.2022.890371

**Published:** 2022-06-06

**Authors:** Zhi-Hai Su, Jin Liu, Min-Sheng Yang, Zi-Yang Chen, Ke You, Jun Shen, Cheng-Jie Huang, Qing-Hao Zhao, En-Qing Liu, Lei Zhao, Qian-Jin Feng, Shu-Mao Pang, Shao-Lin Li, Hai Lu

**Affiliations:** ^1^ Department of Spinal Surgery, The Fifth Affiliated Hospital of Sun Yat-Sen University, Zhuhai, China; ^2^ Department of Radiology, The Fifth Affiliated Hospital of Sun Yat-Sen University, Zhuhai, China; ^3^ Department of Spinal Surgery, The Third Affiliated Hospital of Southern Medical University, Guangzhou, China; ^4^ School of Biomedical Engineering, Guangdong Provincial Key Laboratory of Medical Image Processing, Southern Medical University, Guangzhou, China

**Keywords:** magnetic resonance imaging, diagnosis, deep learning, artificial intelligence, low back pain

## Abstract

**Aim:**

Accurate severity grading of lumbar spine disease by magnetic resonance images (MRIs) plays an important role in selecting appropriate treatment for the disease. However, interpreting these complex MRIs is a repetitive and time-consuming workload for clinicians, especially radiologists. Here, we aim to develop a multi-task classification model based on artificial intelligence for automated grading of lumbar disc herniation (LDH), lumbar central canal stenosis (LCCS) and lumbar nerve roots compression (LNRC) at lumbar axial MRIs.

**Methods:**

Total 15254 lumbar axial T2W MRIs as the internal dataset obtained from the Fifth Affiliated Hospital of Sun Yat-sen University from January 2015 to May 2019 and 1273 axial T2W MRIs as the external test dataset obtained from the Third Affiliated Hospital of Southern Medical University from June 2016 to December 2017 were analyzed in this retrospective study. Two clinicians annotated and graded all MRIs using the three international classification systems. In agreement, these results served as the reference standard; In disagreement, outcomes were adjudicated by an expert surgeon to establish the reference standard. The internal dataset was randomly split into an internal training set (70%), validation set (15%) and test set (15%). The multi-task classification model based on ResNet-50 consists of a backbone network for feature extraction and three fully-connected (FC) networks for classification and performs the classification tasks of LDH, LCCS, and LNRC at lumbar MRIs. Precision, accuracy, sensitivity, specificity, F1 scores, confusion matrices, receiver-operating characteristics and interrater agreement (Gwet k) were utilized to assess the model’s performance on the internal test dataset and external test datasets.

**Results:**

A total of 1115 patients, including 1015 patients from the internal dataset and 100 patients from the external test dataset [mean age, 49 years ± 15 (standard deviation); 543 women], were evaluated in this study. The overall accuracies of grading for LDH, LCCS and LNRC were 84.17% (74.16%), 86.99% (79.65%) and 81.21% (74.16%) respectively on the internal (external) test dataset. Internal and external testing of three spinal diseases showed substantial to the almost perfect agreement (k, 0.67 - 0.85) for the multi-task classification model.

**Conclusion:**

The multi-task classification model has achieved promising performance in the automated grading of LDH, LCCS and LNRC at lumbar axial T2W MRIs.

## Introduction

Low back pain (LBP) is the leading worldwide cause of years lost to disability, and its medical burden is growing alongside the increasing and aging population (1, 2). Lumbar disc herniation (LDH), lumbar central canal stenosis (LCCS) and lumbar nerve roots compression (LNRC) are the most common causes of LBP (1, 3), which are the leading reasons for individuals seeking medical care. Lumbar magnetic resonance (MR) imaging is a crucial tool to explain complicated causes of LBP and decide whether to treat it conservatively or surgically (4–6).

MR imaging is preferred in diagnosing LBP and can accurately grade LDH (7, 8), LCCS (9) and LNRC (10, 11). Each grading of these three diseases plays an essential role in determining appropriate treatment options. However, interpreting these complex MR images (MRIs) is a repetitive and time-consuming workload for radiologists (12). The artificial intelligence-based on deep-learning (DL) algorithm has great potential benefits in medical imaging diagnostics since it can provide semi-automated reports under the supervision of clinicians (13). It may improve the accuracy, consistency, objectivity and efficiency of disease degree assessment, further supporting clinical decision-making. Mathematically, disease diagnosis is a classification problem.

Recently, researchers have proposed some single-task classification models based on the DL algorithm for lumbar disease diagnosis from lumbar spine MRIs (14–16). These models based on the DL algorithm show that they can address this classification problem well with the advantage of automatically learning representative features from MRIs. However, one or more causes of low back pain may coexist on the same axial MRI image. The single-task classification model has the limitation of insufficient information utilization, resulting in missed or delayed diagnosis, which may be limited in clinical application. A multi-task classification model has become one of the current research hotspots to address this challenge. By identifying correlations between multiple training tasks, it carries on joint learning to these tasks, thereby improving the generalization ability of the model. A multi-task classification model for evaluating the severity of numerous lumbar diseases at MRIs would be desirable and help clinicians make a thorough diagnosis.

To the best of our knowledge, few multi-task classification models have been currently developed to classify multiple lumbar diseases at MRIs (17, 18). This study aimed to develop a multi-task classification model that can provide clinicians with a precise and comprehensive diagnostic way for automated grading of LDH, LCCS and LNRC at lumbar axial MRIs. After the model was trained, its accuracy performance was assessed on an internal test dataset and an external test dataset, compared with clinicians.

## Methods

The institutional review board of our institution approved this retrospective study with a waiver of informed consent.

### Datasets Preparation

**Figure 1:**
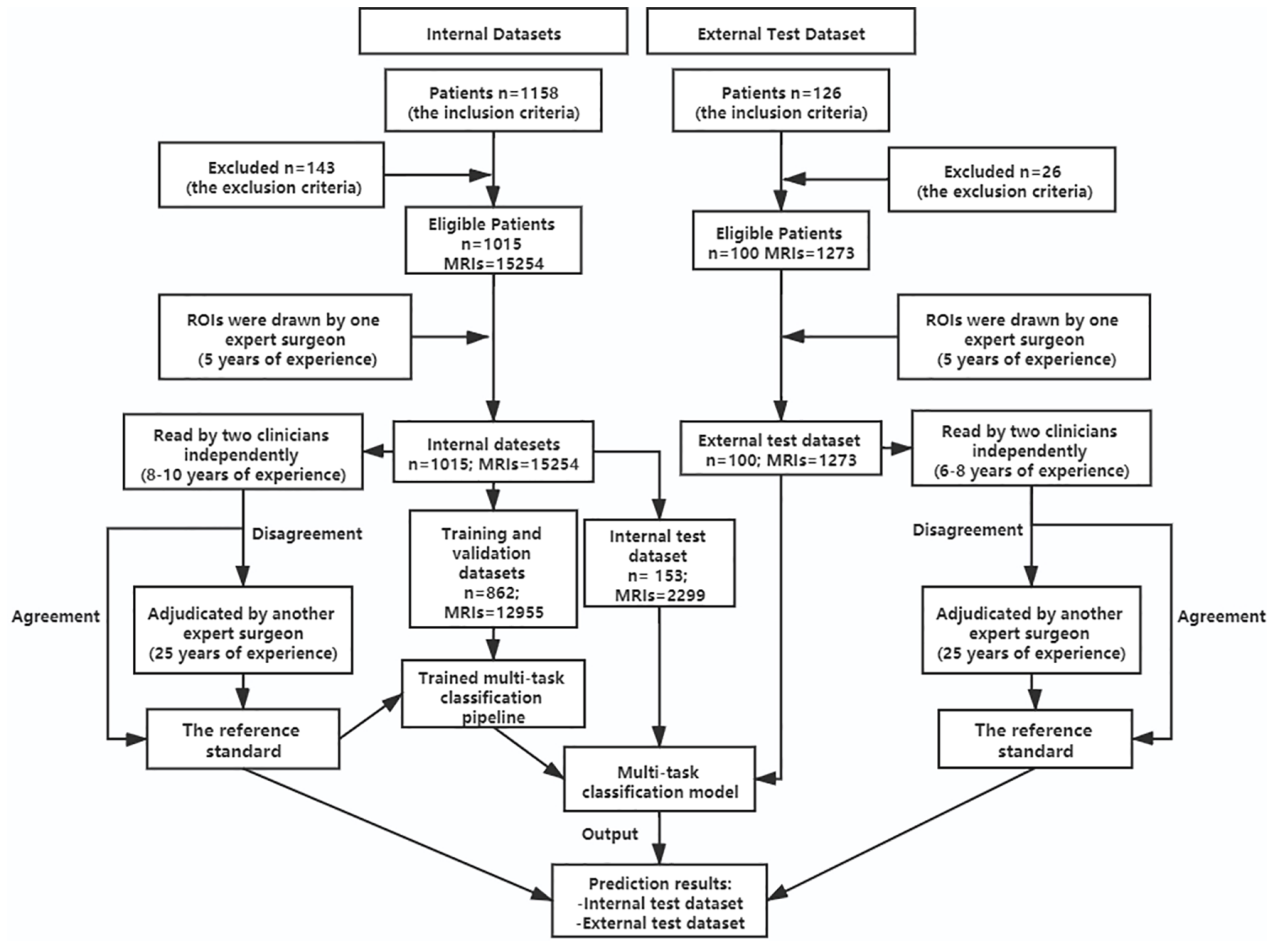
Flowchart showing patients’ selection for the datasets and the process of training multi-task classification model. n, number of patients; MRIs, MRI images; ROIs, regions of interest.

Our study analyzed 15254 axial T2W MRIs as the internal data set collected for 1015 patients who received lumbar spine MRIs in the Fifth affiliated Hospital of Sun Yat-sen University from January 2015 to May 2019. Before, 143 patients were excluded based on the exclusion criteria. We screened studies based on the following inclusion criteria: patients undergoing lumbar MR imaging because of LBP were suitable to participate in this study. Exclusion criteria for the study were as follows: (1) vertebral fractures and/or active inflammation at lumbar MRIs; (2) history of concomitant malignancy; (3) previous spine surgery; (4) severe artifacts at lumbar MRIs. The patients of our internal data set were split into training (n = 710), validation (n = 152) and test sets (n = 153). In addition, external validation was performed on the external test dataset, which contains 1273 axial T2W MRIs from 100 patients who received lumbar spine MRIs in the Third Affiliated Hospital of Southern Medical University, from June 2016 to December 2017. All patients received a lumbar MRI scan using a 3.0-T unit (Magnetom Verio; Siemens, Erlangen, Germany) with T2-weighted turbo spin echo sequence (T2W TSE). The characteristics of T2W TSE in the datasets varied: Repetition time: 3500 to 3775 ms. Echo time: 94 to 120 ms. Field of view: 153 × 153 mm^2^. Slice thickness: 4 to 4.5 mm. Bandwidth: 250 kHz. The lumbar MRI images were stored as DICOM files (Digital Imaging and Communications in Medicine). The patients’ demographics of the two datasets are summarized in **Table 1**, and a flowchart of the data selection processes is illustrated in **Figure 1**.

**Table 1:** Patients’ demographic.

Characteristics	All Internal Datasets (n=1015)	External Test Dataset (n=100)
Age (y)^*^	49 ± 14 (13-88)	53 ± 16 (17-84)
Men	523 (51.5)	49 (49.0)
Women	492 (48.5)	51 (51.0)

Unless otherwise stated, data are numbers of patients, with percentages in parentheses.

^*^Data are means ± standard deviations, with ranges in parentheses.

### Dataset Labeling

As regions of interest (ROIs), Bounding boxes were drawn at all images by one expert surgeon with MRIcro software. Grading on these three lumbar spinal diseases was then performed for this study, with the three classification systems, which were done using well-established criteria for LDH (7, 19), LCCS (9, 20) and LNRC (10, 11).

**Figure 2:**
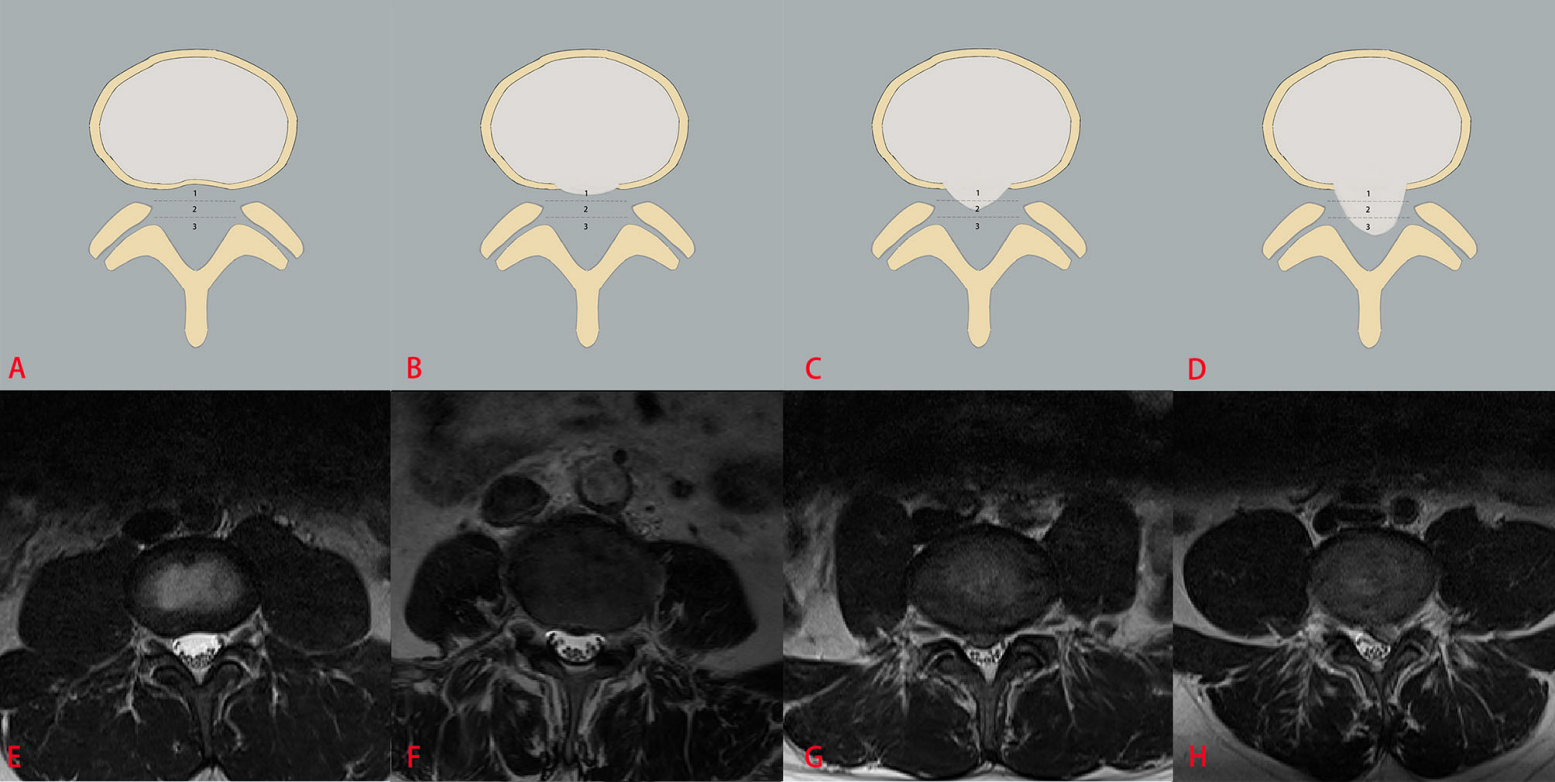
The classification system of the lumbar disc herniation (LDH). The top column is a schematic diagram of four grades with LDH, and the bottom column is the corresponding axial T2W TSE images of four grades with LDH. The size of disc herniation is measured with reference to a single intra-facet line drawn transversely across the lumbar canal, to and from the medial edges of the right and left facet joint articulations. Grade 0 **(A, E)** no disc herniation; Grade 1 **(B, F)** the disc herniation extends up to or less than 50% of the distance from the non-herniated posterior aspect of the disc to the intra-facet line (size-1), Grade 2 **(C, G)** the disc herniation extends up to or more than 50% of the distance from the non-herniated posterior aspect of the disc to the intra-facet line (size-2), Grade 3 **(D, H)**, the herniation extends altogether beyond the intra-facet line (size-3).

**Figure 3:**
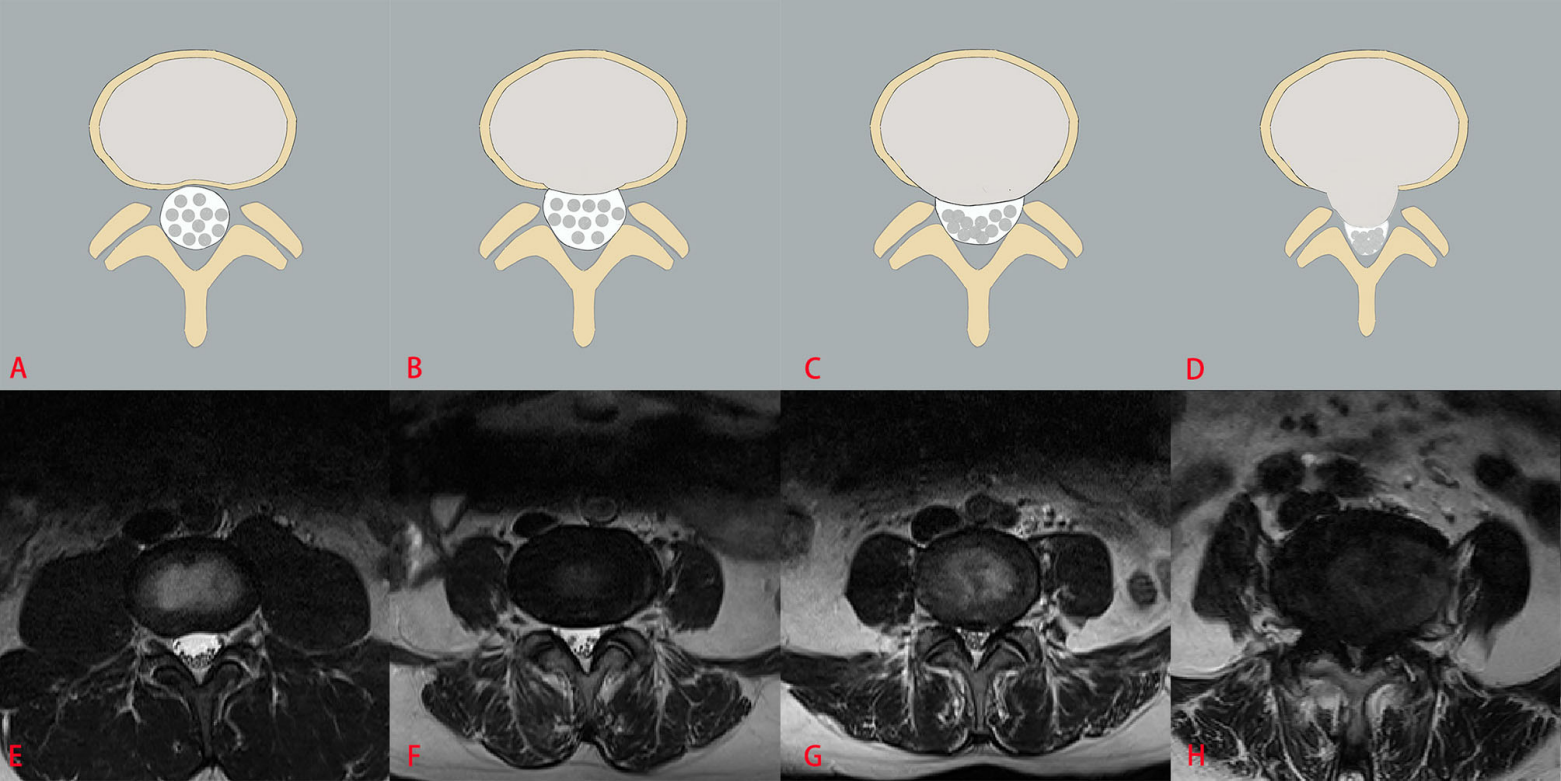
The classification system of lumbar central canal stenosis (LCCS). The top column is a schematic diagram of four grades with LCCS, and the bottom column is the corresponding axial T2W TSE images of four grades with LCCS. LCCS is divided into four grades according to the obliterated severity of anterior cerebrospinal fluid space: Grade 0 **(A, E)** normal; Grade 1 **(B, F)** minor stenosis with clear separation of each cauda equina nerve roots; Grade 2 **(C, G)** moderate stenosis with some cauda equina nerve roots combination; Grade 3 **(D, H)** severe stenosis with all cauda equina nerve roots as a bundle.

**Figure 4:**
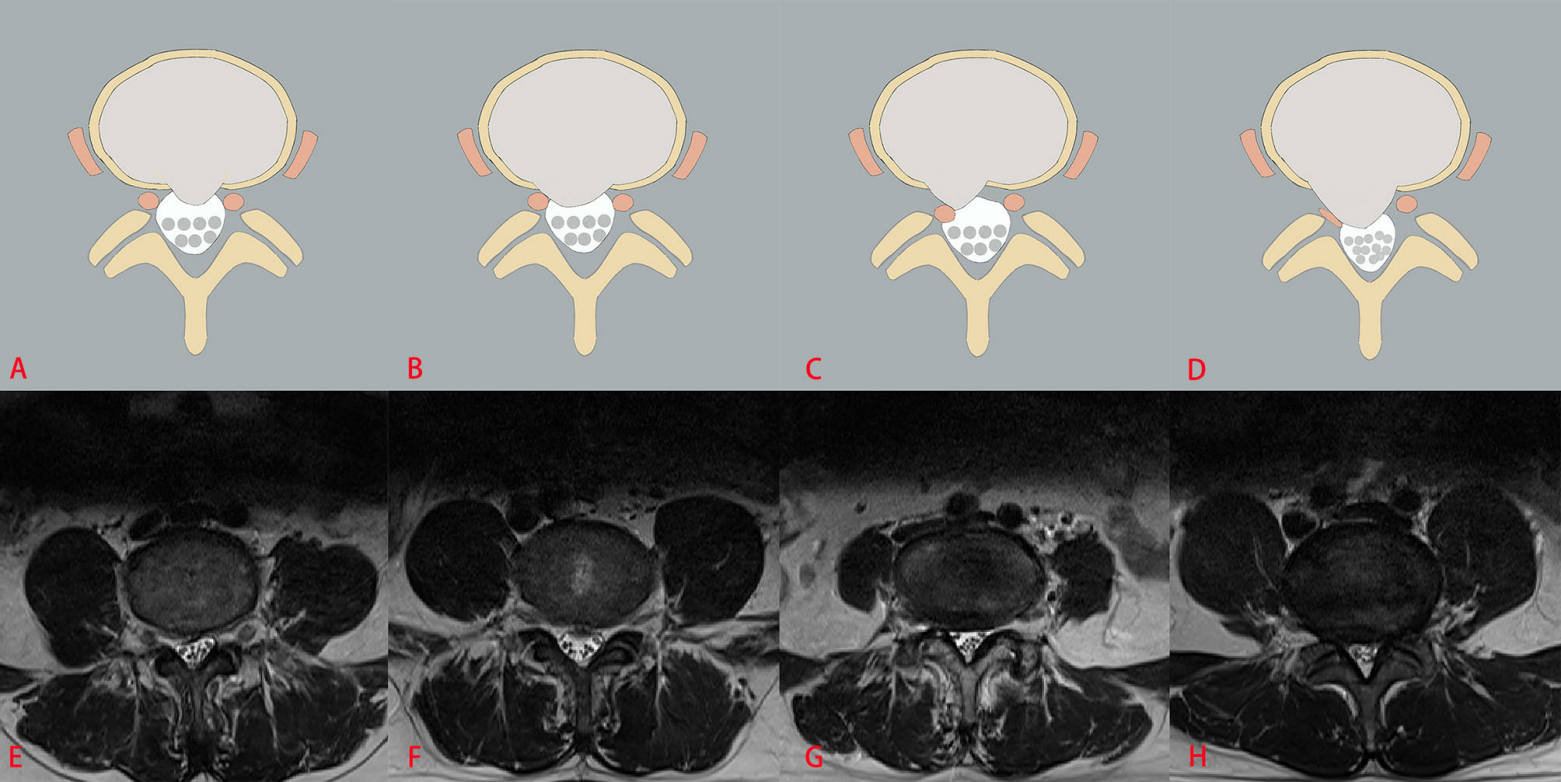
The classification system of lumbar nerve root compromise (LNRC). The top column is a schematic diagram of four grades with LNRC, and the bottom column is the corresponding axial T2W TSE images of four grades with LNRC. Grade 0 **(A, E)** No compromise of the nerve root is seen (normal). There is no evident contact of disk material with the nerve root and the epidural fat layer between the nerve root and the disc material is preserved. Grade 1 **(B, F)** There is visible contact of disc material with the nerve root and the normal epidural fat layer between the two is not evident (contact). The nerve root has a normal position and there is no dorsal deviation. Grade 2 **(C, G)** The nerve root is displaced dorsally by disc material (deviation). Grade 3 **(D, H)** The nerve root is compressed between disc material and the wall of the spinal canal; it may appear flattened or be indistinguishable from disc material (compression).

The three classification systems are described in the following: the classification system of LDH is divided into four grades, according to the size of disc herniation: Grade 0, Grade 1, Grade 2 and Grade 3 (**Figure 2**); the classification system of LCCS is divided into four grades based on the space of anterior cerebrospinal fluid: grade 0, grade 1, grade 2 and grade 3 (**Figure 3**); the classification system of LNRC is also divided into four grades, grade 0, grade 1, grade 2 and grade 3 (**Figure 4**). Two clinicians independently analyzed each axial T2W TSE MR image of the internal dataset and graded it using the above classification systems. In cases of agreement, these grading results of two clinicians served as the reference standard; in cases of disagreement, these grading results were adjudicated by an experienced spinal surgeon to establish the reference standard. Gwet k was used to assess inter-reader reliability between both clinicians for each classification system (21, 22). The external test dataset was graded by two clinicians independently, using the same method. In cases of agreement, these grading results of two clinicians served as the reference standard; in disagreement, these grading results were adjudicated by an experienced spinal surgeon to establish the reference standard.

### Multi-Task Classification Model

**Figure 5:**
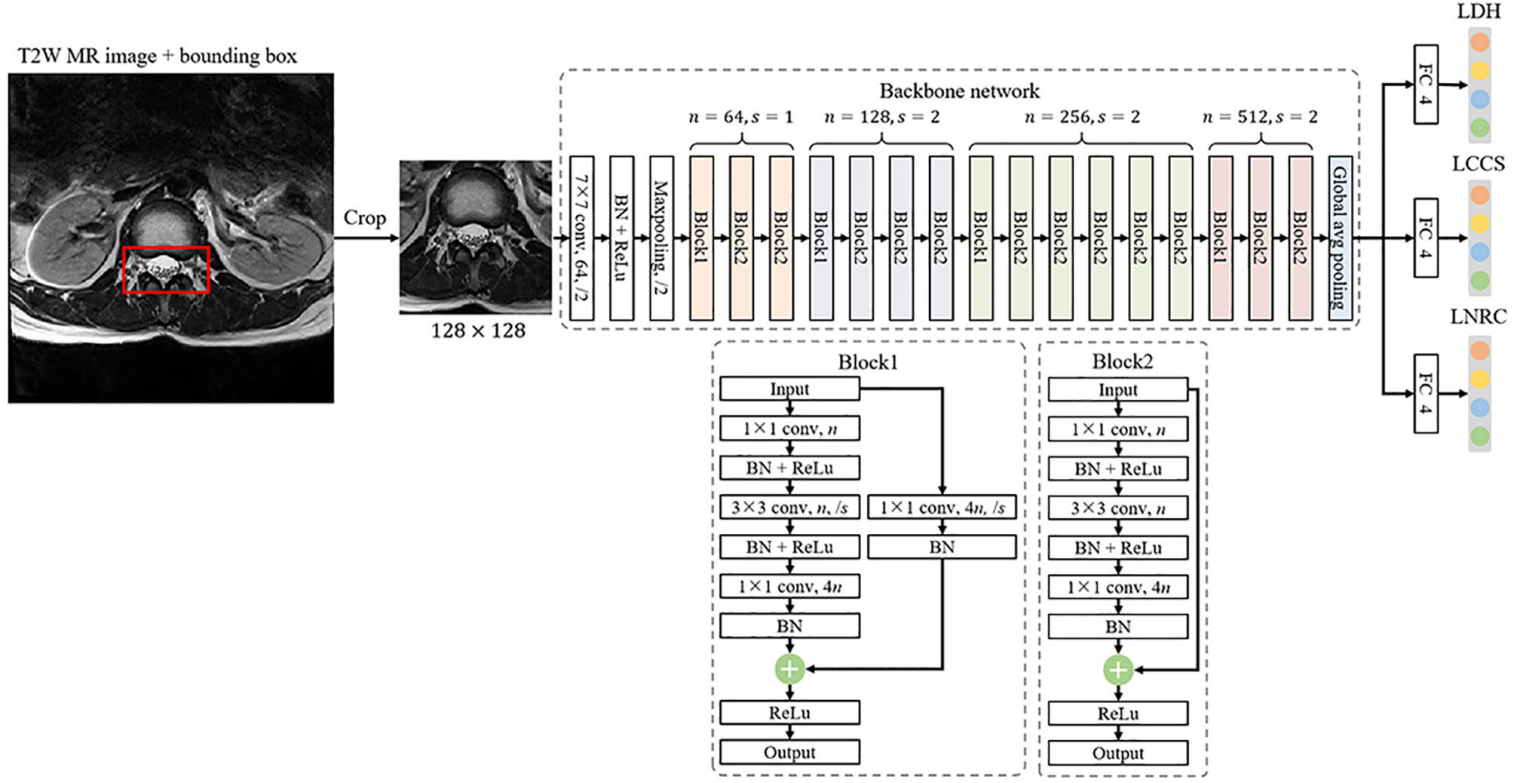
The framework of a multi-task classification network consists of a backbone network for feature extraction and three fully-connected (FC) networks for classification, where n denotes the number of output channels and s is the stride of convolution.

The multi-task classification model consists of a backbone network for feature extraction and three fully-connected (FC) networks for classification, as shown in **Figure 5**. The backbone network is a ResNet-50 framework (23), usually used to extract image features, excluding the fully-connected network. A 2048-dimension feature is extracted by the backbone network and then is put into the three parallel FC networks, whose outputs denote the classification result of LDH, LCCS and LNRC. Cross entropy is used as the loss function. The network is capable of capturing the implicit correlation between LDH, LCCS and LNRC since the three classification tasks share a backbone network. The multitask classification network was trained with a batch size of 16 for 100 epochs using the Adam optimizer with a weight decay of 0.0001. The learning rate was set to 0.001 initially. The training data was online augmented by random rotation of -15° to 15° and random cropping to improve the generalization of the model. All the networks were implemented by Pytorch (https://pytorch.org), and codes ran in a server with an RTX 2080Ti GPU. Training the multitask classification network took about 2.5 hours. Testing an image only took 11 ms.

### Statistical Analysis

Quantitative evaluation metrics including precision, accuracy, sensitivity, specificity, F1 scores, confusion matrices, receiver-operating characteristics (ROC) were applied to assess the diagnostic performance of the multi-task classification network on the two test datasets. Gwet k values with 95% confidence intervals (CIs) were used to evaluate the inter-readers reliability and this model’s clinical reliability. The model was trained and assessed by an information technology engineer.

## Results

### Patient Characteristics in Datasets

A total of 15254 lumbar axial T2W TSE MRIs in 1015 patients in the internal datasets were evaluated. Overall, the mean age of all 1015 patients was 49 years ± 14 (age range, 13-88 years), and the mean age of 492 women was 51 years ± 14 (age range, 15-84 years). (**Table 1**). For the external test dataset, a total of 1273 lumbar spine axial T2W TSE MRIs in 100 patients were evaluated. The mean age of all 100 patients was 53 years ±16 (age range, 17-84 years), and the mean age of 51 women was 56 years ± 15 (age range, 23-84 years). (**Table 1**). The detailed information on reference standard grading at axial MRIs within the two datasets is shown in **Table 2**. The detailed information on reference standard grading from patients within the two datasets is shown in **Supplementary Material**.

**Table 2:** Reference standard classifications of the three lumbar diseases at axial T2W MRIs.

Data Set and Diseases Severity	Lumbar Disc Herniation (LDH)	Lumbar Central Canal Stenosis (LCCS)	Lumbar Nerve Roots Compromise (LNRC)
Internal training and validation dataset			
Grade 0	6450 (49.8)	10770 (93.1)	9403 (72.6)
Grade 1	4933 (38.1)	1836 (14.2)	2227 (17.2)
Grade 2	1435 (11.1)	226 (1.7)	648 (5.0)
Grade 3	137 (1.1)	123 (0.9)	677 (5.2)
Total	12955	12955	12955
Internal test dataset			
Grade 0	1121 (48.8)	1902 (82.7)	1616 (70.3)
Grade 1	864 (37.6)	343 (14.9)	424 (18.4)
Grade 2	287 (12.5)	40 (1.7)	127 (5.5)
Grade 3	27 (1.2)	14 (0.6)	132 (5.7)
Total	2299	2299	2299
External test dataset			
Grade 0	550 (43.2)	1105 (86.8)	866 (68.0)
Grade 1	499 (39.2)	105 (8.2)	305 (24.0)
Grade 2	196 (15.4)	42 (3.3)	44 (3.5)
Grade 3	28 (2.2)	21 (1.6)	58 (4.6)
Total	1273	1273	1273

Unless otherwise stated, data are numbers of patients, with percentages in parentheses.

A high inter-reader agreement with the reference standard was reached on the internal datasets and the external test dataset. For LDH, the k values of the four-grades were 0.93 (95% CI: 0.92, 0.95) for the internal datasets and 0.88 (0.86, 0.90) for the external test dataset. For LCCS, the k values of the four-grades were 0.95 (0.95, 0.96) for the internal dataset and 0.96 (0.94, 0.97) for the external test dataset. For LNRC, the k values of the four-grades were 0.93 (0.92, 0.94) for the internal datasets and 0.91 (0.89, 0.93) for the external test dataset.

### Multi-Task Classification Network Performance on the Internal Test Dataset

**Figure 6:**
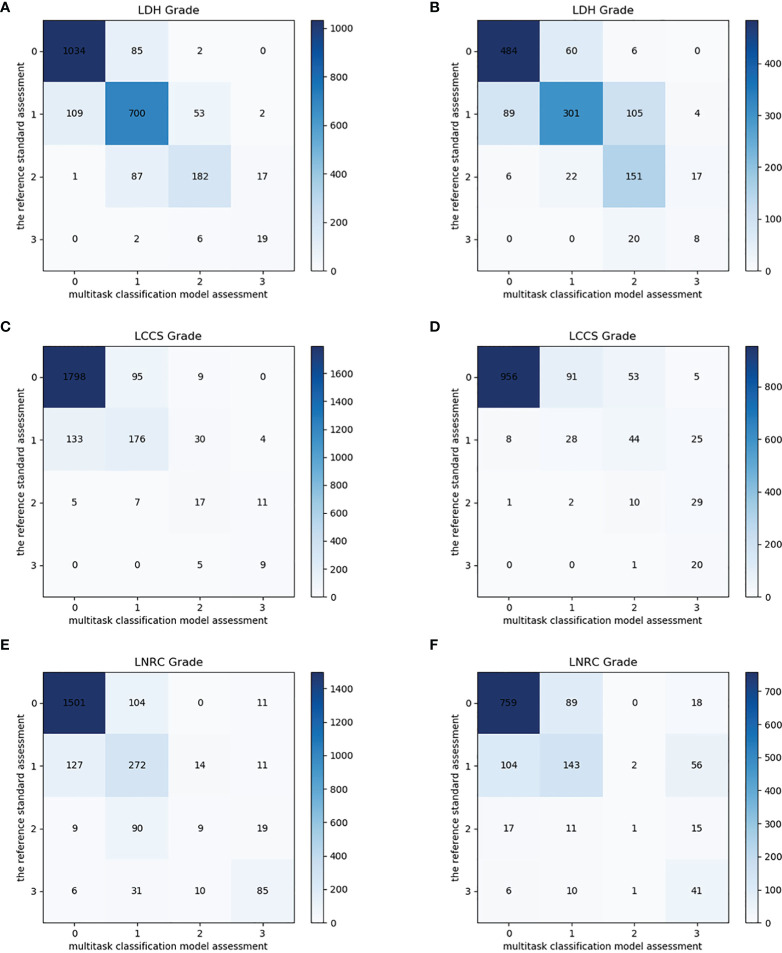
Confusion matrix of the multi-task classification model at axial MRIs. Confusion matrix of the multi-task classification model for grading lumbar disc herniation LDH **(A)**, lumbar central canal stenosis LCCS **(C)** and lumbar nerve roots compression LNRC **(E)** on the internal test dataset. Confusion matrix of the multi-task classification model for grading LDH **(B)**, LCCS **(D)** and LNRC **(F)** on the external test dataset.

**Figure 7:**
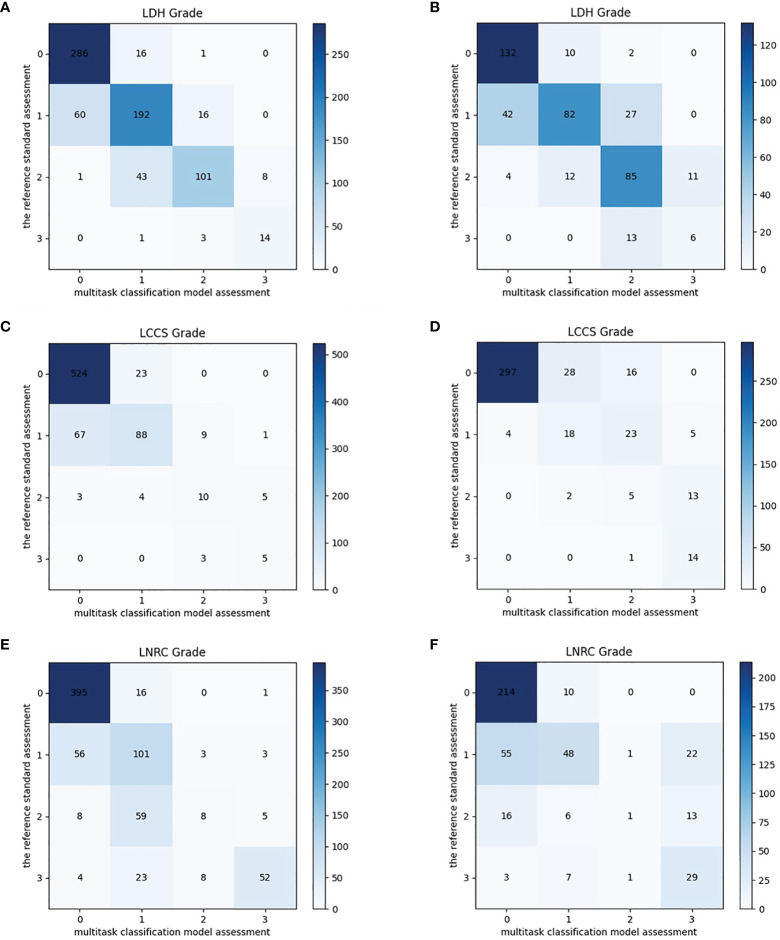
Confusion matrix of the multi-task classification model at lumbar MRI levels. Confusion matrix of the multi-task classification model for grading lumbar disc herniation LDH **(A)**, lumbar central canal stenosis LCCS **(C)** and lumbar nerve roots compression LNRC **(E)** on the internal test dataset. Confusion matrix of the multi-task classification model for grading LDH **(B)**, LCCS **(D)** and LNRC **(F)** on the external test dataset.

**Figure 8:**
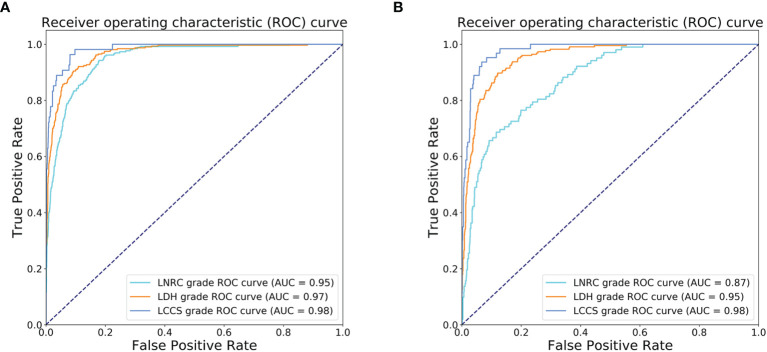
Receiver operating characteristic (ROC) curves of dichotomous grading (grade 0 or grade 1 vs. grade 2 or grade 3) for the three spine diseases. **(A)** ROC curves for the automated grading of three diseases: lumbar disc herniation (LDH), lumbar central canal stenosis (LCCS) and lumbar nerve roots compression (LNRC) on the internal test dataset. **(B)** ROC curves for the automated grading of three diseases: LDH, LCCS and LNRC on the external test dataset.

After training, there was a similar agreement between the multi-task classification model and the reference standard in the internal test dataset, with *k* values of 0.80 (0.78, 0.82) for the four grades of LDH; 0.86 (0.84, 0.87) for the four grades of LCCS; and 0.78 (0.76, 0.80) for the four grades of LNRC. Using the DL algorithm based on ResNet 50 to assess overall grade 0 and the other grades of each classification system, the overall accuracy was as follows: 84.17% (1935 of 2299) for LDH, 86.99% (2000 of 2299) for LCCS, 81.21% (1867 of 2299) for LNRC. Detailed statistics for the four grades of the three classification systems at axial MRIs are summarized in **Figures 6A, C, E**. And detailed statistics for the four grades of the three classification systems at lumbar MRI levels are summarized in **Figures 7A, C, E**. An overview of the results with precision, F1 score, accuracy, k values, sensitivity and specificity are shown in **Table 3**. The area under the ROC curve of the dichotomous classification (grade 0 or grade 1 vs. grade 2 or grade 3) was as follows: 0.97 for LDH, 0.98 for LCCS, 0.95 for LNRC (**Figure 8A**).

**Table 3:** The accuracy performance of the multi-task classification model on the internal test dataset.

Predicted Grading	Precision (%)	F1 Score (%)	Accuracy (%)	Sensitivity (%)	Specificity (%)	Gwet *k*
LDH	84.1	84.1	84.2	90.7	92.2	0.80
Grade 0	90.4	91.3				
Grade 1	80.1	80.6				
Grade 2	74.9	68.7				
Grade 3	50.0	58.5				
LCCS	87.0	86.8	87.0	65.2	94.5	0.85
Grade 0	92.9	93.7				
Grade 1	63.3	56.7				
Grade 2	27.9	33.7				
Grade 3	37.5	47.4				
LNRC	79.7	80.0	81.2	79.2	92.9	0.78
Grade 0	91.4	92.1				
Grade 1	54.7	59.1				
Grade 2	27.3	11.2				
Grade 3	67.5	65.9				

LDH, lumbar disc herniation; LCCS, lumbar central canal stenosis; LNRC, lumbar nerve roots compromise.

### Multi-Task Classification Network Performance on the External Test Dataset

The outstanding robustness of this multi-task classification model was obtained in the external test dataset. Reading grades between the reference standard and the multi-task classification model had a strong correlation with *k* values of 0.67 (0.64, 0.70) for the four grades of LDH; 0.77 (0.75, 0.80) for the four grades of LCCS; and 0.69 (0.66, 0.72) for the four grades of LNRC. The overall accuracy was as follows: 74.16% (944 of 1273) for LDH, 79.65% (1014 of 1273) for LCCS, and 74.16% (944 of 1273) for LNRC. And the F1 scores were 81.3% for LDH, 83.5% for LCCS and 84.2% for LNRC. Detailed statistics for the four-grades of the three classification systems at axial MRIs are summarized in **Figures 6B, D, F**. And detailed statistics for the four grades of the three classification systems at lumbar MRI levels are summarized in **Figures 7B, D, F**. An overview of the results with precision, F1 score, accuracy, k values, sensitivity and specificity are shown in **Table 4**. The area under the ROC curve of the dichotomous grading (grade 0 or grade 1 vs. grade 2 or grade 3) was as follows: 0.95 for LDH, 0.98 for LCCS, 0.87 for LNRC (**Figure 8B**).

**Table 4:** The accuracy performance of the multi-task classification model on the external test dataset.

Predicted Grading	Precision (%)	F1 Score (%)	Accuracy (%)	Sensitivity (%)	Specificity (%)	Gwet *k*
LDH	75.8	74.1	74.2	86.9	88.0	0.67
Grade 0	83.6	85.7				
Grade 1	78.6	68.3				
Grade 2	53.5	63.2				
Grade 3	27.6	28.1				
LCCS	88.6	83.3	79.7	94.6	86.5	0.77
Grade 0	99.1	92.4				
Grade 1	23.1	24.8				
Grade 2	9.1	13.3				
Grade 3	25.3	40.0				
LNRC	74.1	73.4	74.2	68.8	87.6	0.69
Grade 0	85.7	86.6				
Grade 1	56.5	51.3				
Grade 2	25.0	4.2				
Grade 3	31.5	43.6				

LDH, lumbar disc herniation; LCCS, lumbar central canal stenosis; LNRC, lumbar nerve roots compromise.

## Discussion

In this study, we developed and validated a multi-task classification network in the automated grading of LDH, LCCS and LNRC at lumbar axial MRIs. This study is the first comprehensive study of multi-task classification for grading LDH, LCCS and LNRC at axial MRIs. The multi-task classification network demonstrated good performance in the automated grading of LDH, LCCS and LNRC.

MRI plays an important role in the assessment of LBP and accurate grading of LDH (7), LCCS (9) and LNRC (10, 11). However, detailing such assessment information, which may be beneficial to clinicians in providing more effective treatment strategies for patients with LBP, is repetitive and time-consuming and subjective for clinicians (12). A multi-task classification network that reliably classifies the severity of lumbar degenerative diseases may be desired and useful in clinical practice.

Previous studies (14, 24) have shown the potential of the DL algorithm to classify spinal diseases shown on lumbar MRIs. They validated the feasibility of using the DL algorithm to grade LCCS or LDH automatically. However, these automated grading models based on the DL algorithm were only used to classify a single lumbar disease and failed to realize the automated grading of multiple lumbar diseases. Therefore, researchers explored the potential of a multi-task classification network to evaluate multiple lumbar diseases. Jamaludin et al. (25) proposed a multi-task classification network: SpineNet, which was developed for the automated classification of several pathological spinal features, including Pfirrmann grading (26), intervertebral disc stenosis, lumbar central canal stenosis, bone marrow changes and endplate defects. Lu et al. (18) developed the DeepSpine model, which could be used to diagnose lumbar central canal stenosis and neural foraminal stenosis automatically at axial and sagittal T2W MRIs. More recently, in 2021, a multi-task classification model was developed to automatically detect and classify lumbar central canal, neural foraminal stenosis and lateral recess at axial and sagittal T2W MRIs (27). It can be used to evaluate lumbar spinal stenosis in clinical practice quickly. Overall, these studies suggest the efficacy of multi-task classification models in the automated grading of spinal stenosis on lumbar MRIs. To date, Lewandrowski et al. (28) have made a preliminary attempt and demonstrated that a multi-task classification model based on the DL algorithm is feasible for automated grading of disc bulging, disc herniation, and lumbar stenosis at routine MRIs. But they failed to diagnose the severity of disc herniation and lumbar stenosis, which may not be enough to help surgeons make clinical decisions.

The classification systems of these three diseases in our model correlate with surgical treatment modalities. Mysliwiec et al. (7) suggest that patients with grade 1 should be excluded from surgical consideration, and patients with grade 2 or grade 3 should be considered for performing micro discectomies. This classification system is currently widely used in the clinical setting (29, 30). In addition, the classification system of LCCS, a valuable method for clinicians and radiologists to use in clinical practice, is defined by the degree of separation of the cauda equina related to the severity of the clinical signs (9, 31). Moreover, the classification system of LNRC is decided by the spatial relationship between herniated disc material and nerve roots, and this system is proven to be relevant to surgical grading (10, 11). Notably, our model achieved superior performance for the dichotomous classification (grade 0 or grade 1 vs. grade 2 or grade 3). Each result of the area under the ROC curve on the internal test dataset (external test dataset) was as follows: LDH: 0.97 (0.95); LCCS: 0.98 (0.98); and LNRC: 0.95 (0.87). This superior performance demonstrates that our model is a promising tool to be used in clinical practice to assess LDH, LCCS and LNRC.

The model proposed in our study achieves good performance for grading LDH, LCCS and LNRC at axial MRIs. Firstly, on the internal test dataset, our model showed substantial to the almost perfect levels of agreement for the three classification systems with four grades. Especially in the grading of LCCS, our model had a k value of 0.86 for the four grades, which is higher than the k value of 0.82 reported by Hallinan et al. (27). Our model also showed substantial levels of agreement on the external test dataset. In addition, the model has high accuracy for the automated classification of LDH, LCCS and LNRC. The average classification accuracy rates (grade 0, grade 1, grade 2 and grade 3) of LDH, LCCS and LNRC were 84.17%, 86.99% and 81.21%, respectively. Among these, the average classification accuracy of LCCS is higher than that reported in previous studies (14, 18, 27).

Although this multi-task classification network shows considerable consistency and good performance in the automated grading of LDH, LCCS and LNRC, our study has several limitations. First, we selected the international classification systems relevant for surgical selection as the reference standard, although there remains controversy concerning LDH, LCCS and LNRC classification at MRI (31, 32). Second, the diagnostic accuracy of grade 2 and grade 3 for the three lumbar diseases was low in our study, which may be the potential to be associated with a data class imbalance. Increasing the data sample size would be one way to improve the diagnostic accuracy and will be completed in our future work. Using only axial MRIs for grading LDH, LCCS and LNRC may be another limitation, although the grading of three systems is taken from the axial T2W MRIs. In addition, automated grading of multiple lumbar diseases such as Pfirrmann grading and osteoporotic vertebral fractures using multiple MRI sequences should be integrated into our model in the future. Finally, we did not explore the relationship between clinical symptoms and the grading of three systems. This relationship may play an essential role in clinical decision-making and will be completed in our future work.

In conclusion, we proposed a multi-task classification network for automated grading of LDH, LCCS and LNRC at lumbar axial MRIs. The current study found that automated grading of LDH, LCCS and LNRC at lumbar axial MRIs using a multi-task classification network is feasible with moderate to high accuracy. Additionally, our model showed comparable agreement with clinicians in classifying LDH, LCCS and LNRC.

## Data Availability Statement

The original contributions presented in the study are included in the article/**Supplementary Material**. Further inquiries can be directed to the corresponding author.

## Ethics Statement

The studies involving human participants were reviewed and approved by Institutional Review Board of the Fifth Affiliated Hospital of Sun Yat-Sen University. Written informed consent to participate in this study was provided by the participants’ legal guardian/next of kin.

## Author Contributions

Z-HS and JL designed the study, collected and analyzed the data, and revised the manuscript for intellectual content. M-SY and KY conceptualized, analyzed and interpreted the data, and drafted the manuscript. Z-YC analyzed the data. JS and C-JH reanalyzed the data and drafted the manuscript. Q-HZ and E-QL collected and analyzed and interpreted the data. Q-JF contributed to the methods (model training) and revised the manuscript for intellectual content. LZ contributed to the deep-learning algorithms. S-MP analyzed and interpreted the data, developed the multi-task classification model, and drafted and revised the manuscript for intellectual content. S-LL and HL conceptualized and designed the study, interpreted the data, contributed to the discussion, reviewed and edited the manuscript. All authors contributed to the article and approved the submitted version.

## Funding

Study supported by Zhuhai City Innovation and Innovation Team Project, Guangdong Province, China (ZH0406190031PWC), Zhuhai City Industry-University-Research Cooperation Project, Guangdong Province (ZH0406190031PWC), China and supported by the National Natural Science Foundation of China (No. 62001207 and No. 12126603).

## Conflict of Interest

The authors declare that the research was conducted in the absence of any commercial or financial relationships that could be construed as a potential conflict of interest.

## Publisher’s Note

All claims expressed in this article are solely those of the authors and do not necessarily represent those of their affiliated organizations, or those of the publisher, the editors and the reviewers. Any product that may be evaluated in this article, or claim that may be made by its manufacturer, is not guaranteed or endorsed by the publisher.
