# Supplementary material for: Automatic Grading of Disc Herniation, Central Canal Stenosis and Nerve Roots Compression in Lumbar Magnetic Resonance Image Diagnosis
Source: Front Endocrinol (Lausanne). 2022 Jun 6;13:890371. doi: 10.3389/fendo.2022.890371 (PMC9207332; doi:10.3389/fendo.2022.890371)
Supplement: Supplementary file 1 [file Table_1.docx]

| Data Set and Diseases Severity | Lumbar Disc Herniation (LDH) | Lumbar Central Canal Stenosis (LCCS) | Lumbar Nerve Roots Compromise (LNRC) |
| --- | --- | --- | --- |
| Internal training and validation dataset |  |  |  |
| Grade 0 | 5 (0.6) | 277 (32.1) | 64 (7.4) |
| Grade 1 | 312 (36.2) | 467 (54.2) | 275 (31.9) |
| Grade 2 | 464 (53.8) | 62 (7.2) | 227 (26.3) |
| Grade 3 | 81 (9.4) | 56 (6.5) | 296 (34.3) |
| Total | 862 | 862 | 862 |
| Internal test dataset |  |  |  |
| Grade 0 | 0 (0) | 48 (31.4) | 5 (3.3) |
| Grade 1 | 52 (34.0) | 80 (52.3) | 51 (33.3) |
| Grade 2 | 83 (54.2) | 17 (11.1) | 43 (28.1) |
| Grade 3 | 18 (11.8) | 8 (5.2) | 54 (35.3) |
| Total | 153 | 153 | 153 |
| External test dataset |  |  |  |
| Grade 0 | 0 (0) | 34 (34.0) | 3 (3.0) |
| Grade 1 | 20 (20.0) | 36 (36.0) | 42 (42.0) |
| Grade 2 | 62 (62.0) | 15 (15.0) | 20 (20.0) |
| Grade 3 | 18 (18.0) | 5 (5.0) | 35 (35.0) |
| Total | 100 | 100 | 100 |

Table E1: Reference standard classifications of the three lumbar diseases based on patient

Note: unless otherwise stated, data are numbers of patients, with percentages in parentheses.
